# Design and application of an MR reference phantom for multicentre lung imaging trials

**DOI:** 10.1371/journal.pone.0199148

**Published:** 2018-07-05

**Authors:** Simon M. F. Triphan, Jürgen Biederer, Kerstin Burmester, Iven Fellhauer, Claus F. Vogelmeier, Rudolf A. Jörres, Hans-Ulrich Kauczor, Claus P. Heußel, Mark O. Wielpütz, Bertram J. Jobst

**Affiliations:** 1 Department of Diagnostic and Interventional Radiology, University of Heidelberg, Heidelberg, Germany; 2 Translational Lung Research Centre, Heidelberg, Germany; 3 German Centre for Lung Research (DZL), Gießen, Germany; 4 Radiologie Darmstadt, County Hospital Gross-Gerau, Gross-Gerau, Germany; 5 Department of Medicine, Pulmonary and Critical Care Medicine, University Medical Centre Giessen and Marburg, Marburg, Germany; 6 Institute and Outpatient Clinic for Occupational, Social and Environmental Medicine, Ludwig-Maximilians-University, Munich, Germany; 7 Comprehensive Pneumology Centre Munich (CPC-M), Munich, Germany; 8 Department of Diagnostic and Interventional Radiology with Nuclear Medicine, Thoraxklinik at the University of Heidelberg, Heidelberg, Germany; University of Pennsylvania Perelman School of Medicine, UNITED STATES

## Abstract

**Introduction:**

As there is an increasing number of multicentre lung imaging studies with MRI in patients, dedicated reference phantoms are required to allow for the assessment and comparison of image quality in multi-vendor and multi-centre environments. However, appropriate phantoms for this purpose are so far not available commercially. It was therefore the purpose of this project to design and apply a cost-effective and simple to use reference phantom which addresses the specific requirements for imaging the lungs with MRI.

**Methods:**

The phantom was designed to simulate 4 compartments (lung, blood, muscle and fat) which reflect the specific conditions in proton-MRI of the chest. Multiple phantom instances were produced and measured at 15 sites using a contemporary proton-MRI protocol designed for an *in vivo* COPD study at intervals over the course of the study. Measures of signal- and contrast-to-noise ratio, as well as structure and edge depiction were extracted from conventionally acquired images using software written for this purpose.

**Results:**

For the signal to noise ratio, low intra-scanner variability was found with 4.5% in the lung compartment, 4.0% for blood, 3.3% for muscle and 3.7% for fat. The inter-scanner variability was substantially higher, with 41%, 32%, 27% and 32% for the same order of compartments. In addition, measures of structure and edge depiction were found to both vary significantly among several scanner types and among scanners of the same model which were equipped with different gradient systems.

**Conclusion:**

The described reference phantom reproducibly quantified image quality aspects and detected substantial inter-scanner variability in a typical pulmonary multicentre proton MRI study, while variability was greater in lung tissue compared to other tissue types. Accordingly, appropriate reference phantoms can help to detect bias in multicentre *in vivo* study results and could also be used to harmonize equipment or data.

## Introduction

Today, *in vivo* magnetic resonance imaging (MRI) of the lung is increasingly performed in pulmonary diseases such as cystic fibrosis (CF) [1–3], Chronic Obstructive Pulmonary Disease (COPD) [4–7] and chronic thromboembolic pulmonary hypertension [8]. It is also used in epidemiological whole-body MRI studies [9]. In addition, the number of large cohort studies employing computed tomography (CT) or MRI in lung diseases is on the rise. In this context, proton MRI can provide valuable image-based biomarkers (visual scores or quantitative software based metrics) reflecting disease-related structural lung characteristics, such as the extent of airway wall thickening, bronchiectases, mucus plugging, and emphysema, or functional characteristics such as perfusion impairment. A multicentre study design is often inevitable, requiring the use of different scanners leading to heterogeneous image quality. Consequently, the quantification of image quality becomes increasingly important to address potential bias introduced by imaging with different systems.

For lung tissue, little is known about the impact of different MRI scanners on the assessment of morphological and functional aspects. MRI devices from different manufacturers and production series are expected to provide substantial differences in image quality, but there is limited data available on the magnitude of variability in determinants of image quality such as Signal to Noise Ratio (SNR) or Contrast to Noise Ratio (CNR). Since image interpretation in lung MR studies is complicated by poor signal intensity due to the low proton density and inhomogeneity of the magnetic field (B_0_) intrinsic to lung tissue, characteristics of scanner design are likely to have high impact on analysis and interpretation of lung imaging studies [10–12]. Further, multicentre studies may take significant time due to continuous patient recruitment, which presents the additional task of ensuring consistent image quality over the course of the trial with appropriate reference phantoms being scanned at regular intervals as performed, for example in the COPDgene study [13] or in the European cystic fibrosis clinical trials network (SCIFI CF) [14]. Generally, such variability cannot be fully compensated by calibration and could therefore influence the interpretation of study results, for example when correlating imaging biomarkers with other clinical tests.

While recent CT-based studies on COPD [13] or CF [1] applied commercial reference phantoms, appropriate phantoms for lung MRI are not yet commercially available. Reference phantoms designed for the quality assurance of MRI studies have, until now, mainly been developed with focus on cerebral imaging, along with software which provides fully automated image processing [15–19], especially for functional MRI (fMRI) [20]. However, none have been designed with the challenges of proton lung MRI studies in mind, such as low proton density and local magnetic field inhomogeneities, due to the rarity of lung imaging in MRI. Besides, varying capabilities of different MRI scanners in the context of lung imaging have not been investigated systematically before. Consequently, the aim of this study was to build a cost-effective and simple to use reference phantom specifically designed for proton MRI of the lung. In addition, this is the first phantom study to comparatively assess intra- and inter-scanner variability of SNR and CNR of different structural and functional proton-MRI sequences for current lung MRI in 15 different MRI scanners.

## Methods

### Prerequisites

The available and suitable scanners for lung MRI [21] span a range of available models and differ in B_0_ field strength (1.5T and 3.0T), gradient strength (between 33mT/m and 45mT/m), signal reception hardware (both the number of reception coils and receive amplifiers) as well as length and width of the magnet bore. The bore dimensions, both length and diameter, have a significant effect on the B_0_ field homogeneity at a distance to the isocentre, which in turn causes distortions. Due to the magnetic field inhomogeneities in the lungs, which also lead to very short T2* relaxation time, B_0_ is additionally important, while the gradient amplitudes available are mainly relevant since they determine the available echo time (TE) and thus the degree of T2*-weighting [22, 23]. Finally, due to the large field of view required for lung imaging, the size of the bore is notable since it determines the extent of distortions at the fringes of images.

A primary lung MRI protocol (with essential parameters shown in Table 1) was designed for the *in vivo* study based on a clinical routine protocol optimized to deliver the best achievable diagnostic information on lung structure and function in COPD patients [3, 24]. This was then adjusted by the coordinating centre for each scanner hardware configuration, varying in parameters including resolution, echo time, repetition time (TR) as well as the degree of acceleration by parallel imaging. Since SNR and measurement time are so limited in lung imaging, it was necessary to optimize sequences with regard to diagnostic quality, in general on the cost of comparability between scanner models and software versions. Parameters were also chosen to minimize the number and length of breath-holds for patients, exploiting the capabilities of each scanner. However, these adjustments were kept as small as possible and the same protocol was used on identical scanner configurations. This approach is currently used in most multicentre lung imaging studies using 1H-MRI techniques, since due to the limited SNR, it is mandatory to produce as many diagnostically useful images as possible instead of maximizing effective inter-scanner variability on the cost of SNR. In this context, it is not useful to adjust scan parameters on all available scanners to match image quality of the least capable scanners. As such, the phantom measurements shown here reflect both differences in scanner hardware directly as well as the sequence parameters applicable due to that hardware.

**Table 1:** Sequence parameters of the study protocol used on the Siemens Aera Scanners for *in vivo* measurements and phantom measurements.

Sequence unit	md	or	TR ms	TE ms	FoV mm^2^	d mm	voxel size mm^2^	matrix	pf	TA min:s
VIBE	3D	cor	3.61	1.63	400×400	4.0	1.39×1.39	288×288	2	0:16
VIBE	3D	tra	3.29	1.61	400×300	4.0	1.25×1.25	320×240	2	0:16
HASTE	2D	cor	314.0	20.0	400×400	6.0	0.78×0.78	512×512	3	0:13
HASTE	2D	tra	500.0	27.0	450×366	8.0	1.41×1.41	320×260	2	0:35
TrueFISP	2D	cor	448.9	1.17	400×400	4.5	0.78×0.78	512×512	3	2:20
BLADE	2D	cor	905.0	73.0	400×400	6.0	1.25×1.25	320×320	2	2:13
HASTE IRM	2D	tra	502.0	72.0	400×400	6.0	1.56×1.56	256×256	2	0:38
Angio	3D	cor	2.80	1.04	350×400	1.8	1.04×1.04	336×384	3	0:16
TWIST	3D	cor	1.73	0.76	366×450	5.0	1.76×1.76	208×256	2	0:37
VIBE FS	3D	tra	3.29	1.61	400×300	4.0	1.25×1.25	320×240	2	0:17

Shown are excitation mode, slice/slab orientation, repetition time, echo time, field of view, voxel size, image matrix, parallel imaging factor and total acquisition time. Note that the *in vivo* study contains additional repetitions of identical sequences in different respiratory states as well as after contrast agent injection.

### Phantom design and fabrication

Several requirements were defined for the proposed phantom: To allow for manufacturing a number of phantoms and sending one to each study centre, any single instance should be sufficiently cheap to produce as well as small enough for regular mail transport. For the phantom measurements to be practical, they would have to be performed by the trained technicians on-site using the original protocol designed for the study and be appropriately simple to handle. Thus, both differences in scanner hard- and software as well as the protocol parameters would be considered as they affect patient images. Tissue categories encountered in lung imaging should be emulated by the phantom, especially the lung parenchyma.

**Fig 1:**
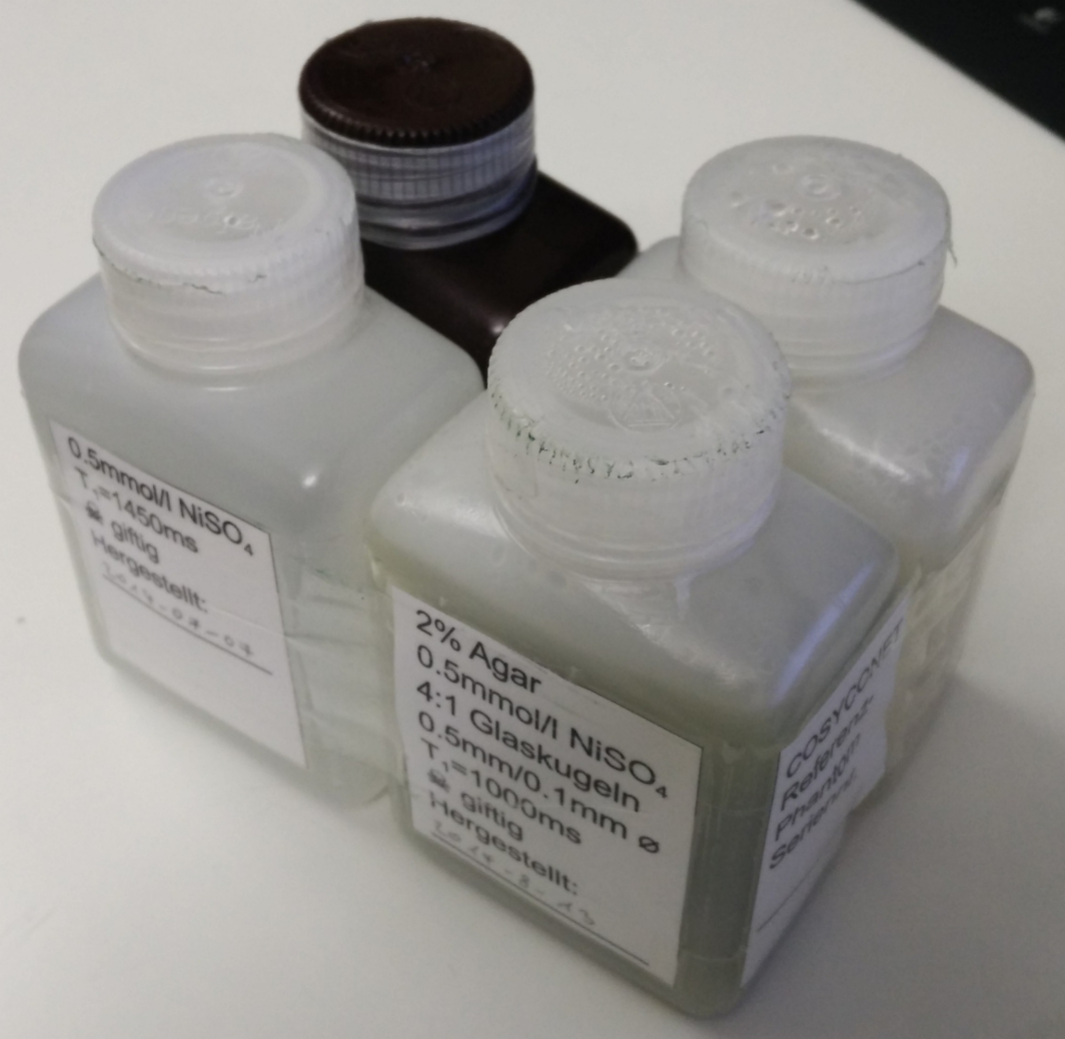
Photo of a phantom instance.

The phantom was chosen to simulate four tissue groups significant for lung imaging: Blood, muscle/thoracic walls, fat and the lung parenchyma itself. Compartments for each phantom instance were produced using rectangular 250ml HDPE bottles, measuring 114×76×48mm, as shown in Fig 1.

For the blood compartment, a solution of 0.5mmol/l NiSO_4_ was intended to provide a T1 relaxation time similar to blood while also allowing for liquid consistency. For the muscle/thoracic wall, a 4% agar-agar preparation with 1.2mmol/l NiSO_4_ was used. Once the agar cools after having been heated to above 95°C, this results in a gelatinous material, which was again adjusted for T1 using NiSO_4_. This was cast around a 6mm diameter acrylic rod placed diagonally inside the phantom bottle to provide a structural element with reproducible position and size within the phantom.

The fat compartment contains canola (rapeseed) oil, which was closest in T1 to the desired value, even though this means the fat in the phantom is liquid, unlike *in vivo*. This was chosen since the study protocol includes fat suppression using an inversion recovery STIR-sequence as well as gradient echo sequences that utilize spectral fat saturation.

To have some way to estimate contrast- and signal-to-noise ratios in the lung parenchyma, a phantom compartment that emulates the high inhomogeneity of the magnetic susceptibility and low proton density in the lung is required. To approximate the structure of alveoli, which have a diameter of about 200μm, a mixture of 500μm and 100μm diameter glass beads at a mass ratio of 4:1 was used. On these, a mixture of heated 2% agar-agar and 0.5mmol/l NiSO_4_ is poured and stirred thoroughly. Since the glass beads do not have a uniform size, they would separate due to size segregation when shaken if the surrounding solution were fluid [25, 26], which is prevented by the agar. Further, in order to reduce the amount of air bubbles in the resultant gel, it was set up to cool slowly by placing the bottles in a water bath heated to 60°C-80°C, giving bubbles time to rise out of the gel. As for the blood and muscle compartments, the concentration of NiSO_4_ was chosen to achieve a T1 comparable to lung tissue in COPD [27]. Each bottle was labelled according to its contents, with a ‘poison’-notice if it contains NiSO_4_ and the date of its fabrication or filling. For each phantom instance, one of each compartment was manufactured and taped together in identical configuration.

The present study was solely based on phantom measurements and did not involve human participants, specimens or tissue samples, or animals, embryos or tissues. Therefore, the study does not require ethical approval.

### Measurements

To verify the appropriateness of the phantom design, T2* in the compartments of one phantom instance was measured using a 2D ultra-short TE (UTE) multi-echo sequence [28]: Signal was acquired at TE1-5 = 70μs, 0.50ms, 1.20ms, 1.65ms, 2.30ms, 9.53ms, 14.3ms and fitted to an exponential decay function. T1 was quantified using a radial look-locker inversion recovery sequence [29] with temporal resolution 18.6ms and 3s maximum inversion time.

At 15 centres, four different scanner models from the same vendor (Siemens Medical, Erlangen, Germany) were used: Avanto, Aera, Espree and Trio. Aera, Avanto and Espree provide main fields of B_0_ = 1.5Tand the Trio has B_0_ = 3.0T. While the only relevant difference between the Avanto (d_B,Avanto_ = 60cm, l_B,Avanto_ = 150cm) and Espree (d_B,Espree_ = 70cm, l_B,Espree_ = 120cm) is that the Espree’s bore is wider and shorter, the Aera (d_B,Aera_ = 70cm, l_B,Aera_ = 137cm) has bore dimensions between the two and a more advanced set of receive coil arrays with a larger number of coils (e.g. body arrays with 18 rather than 6 coils, spine arrays with 32 rather than 18 coils). Also, the Aera scanners and one Avanto use newer generation software (VD) than the others (VB), which differs with regards to image acquisition and reconstruction. Accordingly, the parallel imaging capabilities vary significantly between these two groups of scanners and two slightly different sets of protocol parameters were chosen for the *in vivo* study.

For each of these scanner models (except the Trio), there is one version with a gradient system capable of 33mT/m and one with 45mT/m in use, which, in the case of the Avanto systems, was also equipped with a receive amplifier with a lower number of channels.

The imaging protocol for the *in vivo* study was designed to provide diagnostic information on lung structure and function, similar to protocols in cystic fibrosis, but optimized for COPD patients [24]. As such, it includes sequences of several types: T1-weighted gradient echo sequences (VIBE) with minimal echo times, half-Fourier turbo spin echoes (HASTE), T2-weighted radial TSE (BLADE), balanced steady state free precession (TrueFISP) and additional gradient echo sequences intended for angiography and for dynamic perfusion quantification (TWIST). While most of these sequences are acquired in coronal orientation, the VIBE and HASTE are repeated in transverse orientations with near-identical parameters. Finally, two different types of fat-suppressed methods are employed: A transverse HASTE image using short TI inversion recovery (STIR) and transverse VIBE using fat saturation pulses. Except for the TrueFISP, all sequences are acquired during breath-holds *in vivo* and thus have acquisition times shorter than 20s or are split into several such acquisitions.

In the *in vivo* study, the most relevant biomarkers derived from the above mentioned MRI acquisitions were visual scores (3-point rating scale, lobe based assessment) for 1) the extent of airway wall thickening/bronchiectasis, mainly based on pre-contrast VIBE sequences in coronal and transverse orientation 2) the extent of parenchymal defects (emphysema) based on structural MRI sequences (mainly HASTE sequences in coronal and transverse orientation), and 3) the extent of perfusion defects (visually perceivable defects at time point of peak enhancement in subtracted coronal TWIST images) as well as quantitative software-based perfusion parameters (pulmonary blood flow (PBF), pulmonary blood volume (PBV), mean transit time (MTT)). Other visual biomarkers are the extent of mucus plugging (HASTE, coronal and transversal) and the extent of peribronchial nodules reflecting bronchilitis (HASTE and contrast enhanced VIBE, each cor + trans). Each of these biomarkers is assessed semi-quantitatively with a visual 3-point rating scale. The BLADE sequence serves as alternative in case the HASTE sequences are unusable due to artifacts. The HASTE irm provides T2 weighted images with fs, and is mainly used to further characterize incidental findings in synopsis with the other sequences. The Angio FLASH sequence is used to exclude pulmonary embolism, which is mandatory when interpreting the dynamic TWIST perfusion study. The free-breathing TrueFISP was added to the protocol since it is robust to motion artifacts and allows to estimate respiratory mechanics.

For the phantom measurements, the intended breathing state is of course irrelevant and each measurement was performed with 5s delays in between to ensure relaxation. Just as the patient measurements in the *in vivo* trial are necessarily performed by different teams of technicians at each site, phantom measurements were also completed by the local technicians according to precise instructions provided together with the phantom instances. Phantom measurements were acquired at baseline of the *in vivo* study, and, as far as possible, repeated every 3 months for as long as patient data was acquired at each individual site, resulting in between 1 and 5 phantom measurements each.

The parameters in use both for the *in vivo* study and the phantom measurements given in this work are shown in Table 1. Note that these are given for the measurements on Aera scanners and that the parameters used on the other systems were slightly different. These choices were necessary to optimize for image quality in the lungs, while adapting to the different gradient and main field strengths as well as the receive coils available at each study centre as mentioned above.

### Image data analysis

Image parameters were determined from a set of MR images measured using the unmodified base protocol for the multicentre study, removing only automated breathing commands. The images were processed using custom software written in Python for this purpose. All parameters were evaluated separately for each MR sequence.

**Fig 2:**
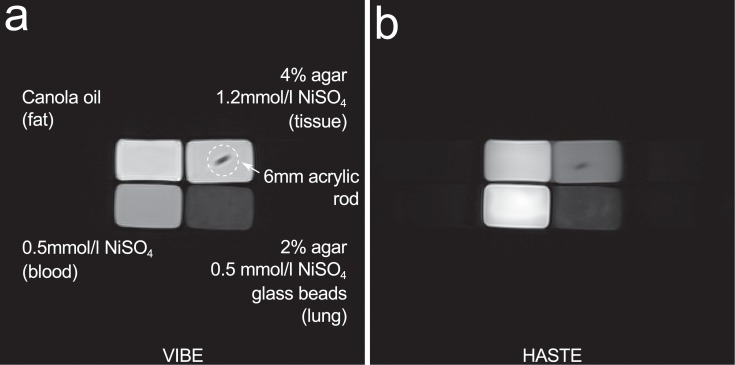
Gradient echo and turbo spin echo MR images of the phantom. a: A single, central slice of a 3D VIBE acquisition showing all phantom compartments. b: A slice acquired using HASTE. Slices are equivalent to coronal orientation.

Since it provides comparatively high SNR and resolution at minimal sensitivity to artefacts, the first step of locating the phantom bottles in the images was completed in the first image series, which is a coronal GRE. One annotated slice of this is shown in Fig 2a. The phantom bottles are detected using a watershed [30] on the sum of all slices of the 3D acquisition, with the bottom of the bottles determined from a second transverse projection using a simple threshold. The top of each compartment is finally cut off exploiting the known dimensions of the bottles, since the uppermost layer in the lung-like compartment tended to be inhomogeneous due to the fabrication procedure. Since the lung compartment was made of glass beads of different sizes being fixed in agar containing NiSO4, a small amount of supernatant mainly containing agar developed above the glass bead layer during the cooling and solidification process. This layer does not have the attributes of T2* and proton density desired for the simulation of lung parenchyma. Due to T1-weighting, compartments were easy to identify by their relative signals in the GRE image, giving an association of each voxel to a specific compartment (or background). For the images of all other sequences, this 3D association map was used by deforming and transposing it according to the position and pixel spacing information taken from DICOM tags of both images.

While this association map provides signal amplitudes for each compartment and sequence, signal-to-noise and contrast-to-noise ratios require a measure of noise for normalization. Unfortunately, in the available DICOM images, noise is highly inhomogeneously distributed and cannot be properly quantified in the outward regions of the images since they are stored as 12-bit integers, which results in background areas consisting in large parts of just zeroes and ones. Thus, to provide a reasonable estimate of noise, a simple 3-dimensional edge detection kernel was convolved onto the image to determine local variations. All voxels above a threshold in this convolved image were considered to be actual edges in the original image, while zeroes were assumed to be part of areas with undetectable noise due to the integer representation. Ignoring these voxels, the standard deviation σ_n_ of the remaining voxels in the convolved images was used as noise estimate and used together with the association map to calculate an SNR value for each sequence and compartment. CNR values for each sequence and pair of compartments were determined from the signal difference divided by σ_n_.

Two separate measures were implemented to reflect structural depiction: The shape of the acrylic rod as it appears in images and the reproduction of the phantom bottles’ edges. For the rod depiction, the rods position is first determined by detecting contours below a threshold in the already found ‘muscle’ compartment. Since the rod is always straight, its orientation can then be identified using a simple linear fit. From this, the signal gradients orthogonal and parallel to the rod’s orientation projected into the imaging slice are determined. These each correspond to the representation of the rod cross section within the slice and the slice profile, respectively. To reduce these curves to a single number, the Full Width at Half Maximum (FWHM) is calculated.

Additionally, the edge representation of each sequence is determined in the two orthogonal directions within each image set. To do so, the centre of each compartment is calculated from its centre of mass and the signal gradient orthogonal to the two outside edges of the bottle, starting from this centre, is averaged in a small strip. Again, a single number for each direction is achieved by fitting a sigmoidal function to this gradient and using the width of the sigmoid as the measure of edge blurring.

### Statistics

The results below are given as mean ± standard deviation unless otherwise stated. As measures of repeatability, standard deviations between the quality measures determined from multiple measurements were computed. To examine the statistical significance of the differences found between the scanner models in use, p-values were calculated using the Mann-Whitney U test, whereby p-values below 0.05 were considered significant.

## Results

According to the UTE measurement, the transverse relaxation times in the phantom were T2*_L_ = (2.05±0.26)ms for the lung compartment and T2*_F_ = (10.00±0.16)ms, T2*_M_ = (32.5±0.59)ms, T2*_B_>100ms for the fat, muscle and blood compartments, respectively. While this T2* for the lung compartment is still longer than what has been found *in vivo* (1.5ms, [28]), this was considered sufficiently short to replicate susceptibility effects.

The T1 measurement gave T1_L,F,M,B_ = (871±41)ms, (183±6)ms, (762±13)ms, (1377±23)ms for each compartment. T1 in the lung compartment is still somewhat shorter than what has been found in the lungs of COPD patients previously [31]. However, lung T1 strongly depends on the state of the lungs and thus is generally lower in COPD patients with worse disease and varies strongly between patients. Accordingly, this was considered acceptable. Notably, while producing shorter T1 is easily possible, providing both longer T1 and maintaining the desired T2* is difficult.

For short-term intra-scanner variability, repeated measurements (n = 5) of the same phantom on the same scanner unit provided low relative standard deviations in SNR for each compartment, in particular 4.5% for lung tissue, 4.0% for blood, 3.3% for muscle, and 3.7% for fat.

A total of 12 phantom instances was produced, and a certain inter-instance variability had to be expected since all instances were hand-made prototypes. Thus, inter-instance variability had to be assessed. In this context, the comparative measurements of the 12 phantom instances on the same scanner revealed an average relative standard deviation in SNR of 12.2% for the lung compartment, 3.2% for blood, 6.7% for muscle and (excluding bSSFP, which exhibits characteristic banding artefacts and fat-suppressed sequences, where SNR for fat is purposely minimal) 2.7% for fat.

Measuring all 15 scanners using 12 phantom instances (at baseline of the *in vivo* study), SNR values ranged from 1.360±0.055 for the lung compartment in the Angio FLASH sequence to 174.0±3.6 for the blood compartment in the transversal HASTE sequence. Table 2 shows SNR averaged over the mean in the baseline measurements as well as the standard deviation between these values are for all sequences in the study protocol.

**Table 2:** Mean and standard deviation of SNR over all 15 scanners measured at baseline of the *in vivo* study using 12 phantom instances, ordered by sequence and phantom compartment.

Sequence	Lung	Blood	Muscle	Fat
VIBE cor	6.1±3.5	30.3±13.2	39.6±18.4	49.9±24.3
VIBE tra	6.5±3.3	33.3±12.3	42.1±16.7	52.8±20.3
HASTE cor	18.7±9.4	215.4±85.3	81.4±28.2	166.2±60.7
HASTE tra	26.5±13.7	234.6±99.1	99.4±41.4	176.6±74.5
TrueFISP	4.4±2.7	112.3±55.6	32.3±20.6	58.8±24.9
BLADE	3.0±1.7	118.2±81.0	12.3±3.9	75.7±24.2
HASTE IRM	4.8±2.5	125.5±30.5	15.7±6.8	4.4±2.3
Angio	1.2±0.3	2.8±1.6	4.9±2.7	15.4±3.6
TWIST	3.0±2.2	8.5±5.2	13.6±8.0	51.7±32.7
VIBE FS	3.9±2.0	29.9±8.7	39.4±15.2	12.6±7.9

When we performed the initial phantom measurements across all 15 imaging sites (using 12 phantom instances) upon the start of the *in vivo* study, the inter-scanner-variability in SNR was found to be significantly larger than the abovementioned short-term intra-scanner and also inter-instance variability. For example, in the BLADE sequence, inter-scanner variability ranged from 7% in the fat compartment to 56% in the blood compartment. In general, for most of the sequences in the study protocol, the lung compartment provides the largest inter-scanner variation, between 31% in the coronal HASTE and 62% in the TWIST (disregarding the Angio FLASH sequence which has too little SNR to be reasonably compared). Overall, the average relative difference in SNR to the median was 41% for the lung compartment, 32% for blood, 27% for muscle and 32% for fat.

**Fig 3:**
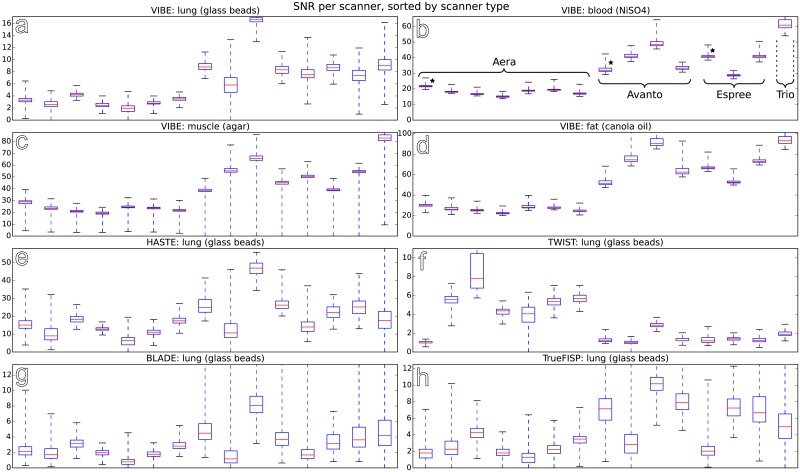
SNR obtained from all 15 scanner units (using 12 phantom instances) for the coronal VIBE sequence, sorted by MR scanner type and phantom compartment (a-d), as well as SNR for the HASTE, TWIST, BLADE and TrueFISP sequences, given for the lung compartment only (e-h). Scanners using 33mT/m gradient systems are marked using stars. Please note that only the initial phantom measurements acquired at baseline of the multicentre *in vivo* study are shown. Data from follow-up measurements performed at 3 month intervals were left out to maintain clarity.

Fig 3 shows the quartiles of the SNR found for the coronal VIBE sequence in all compartments as well as SNR in the lung compartment only for the HASTE, TWIST, TrueFISP and BLADE sequence. Note that values are not only sorted by scanner types, but that for all types except the Trio there is one device with a weaker gradient system. While longitudinal measurements were acquired for each study site at 3 month intervals, only the baseline measurements are shown here to ensure clarity and comprehensibility.

Several follow-up measurements at 3 month intervals were available for most scanner units, providing 3–5 measurements for each of the Aera, 2–5 for the Avanto, 2–6 for the Espree systems, and 3 for the Trio system. When considering all available measurements (i.e. initial phantom measurements across all 15 imaging sites at baseline of the *in vivo* study, and additional follow-up measurements), the differences in SNR apparent in Fig 3 can be examined statistically: Comparing SNR in the VIBE sequence and the lung-equivalent compartment yields p<10^−4^ for the differences between the Aera systems and each of the Avanto, Espree and Trio groups. Conversely, among the Avanto, Espree and Trio groups, the test gives p>0.05. While the 3T system, the Trio tends to provide notably different values than the 1.5T systems in several parameters, the divergence is not statistically significant due to the small number of measurements available on the 3T system.

Comparing the otherwise identical T1-weighted sequences with and without fat suppression pulses, the suppression effectiveness given as the relative reduction of signal was found to be 87.0%±3.7% on average, reflecting mainly the local B_0_ homogeneity.

**Fig 4:**
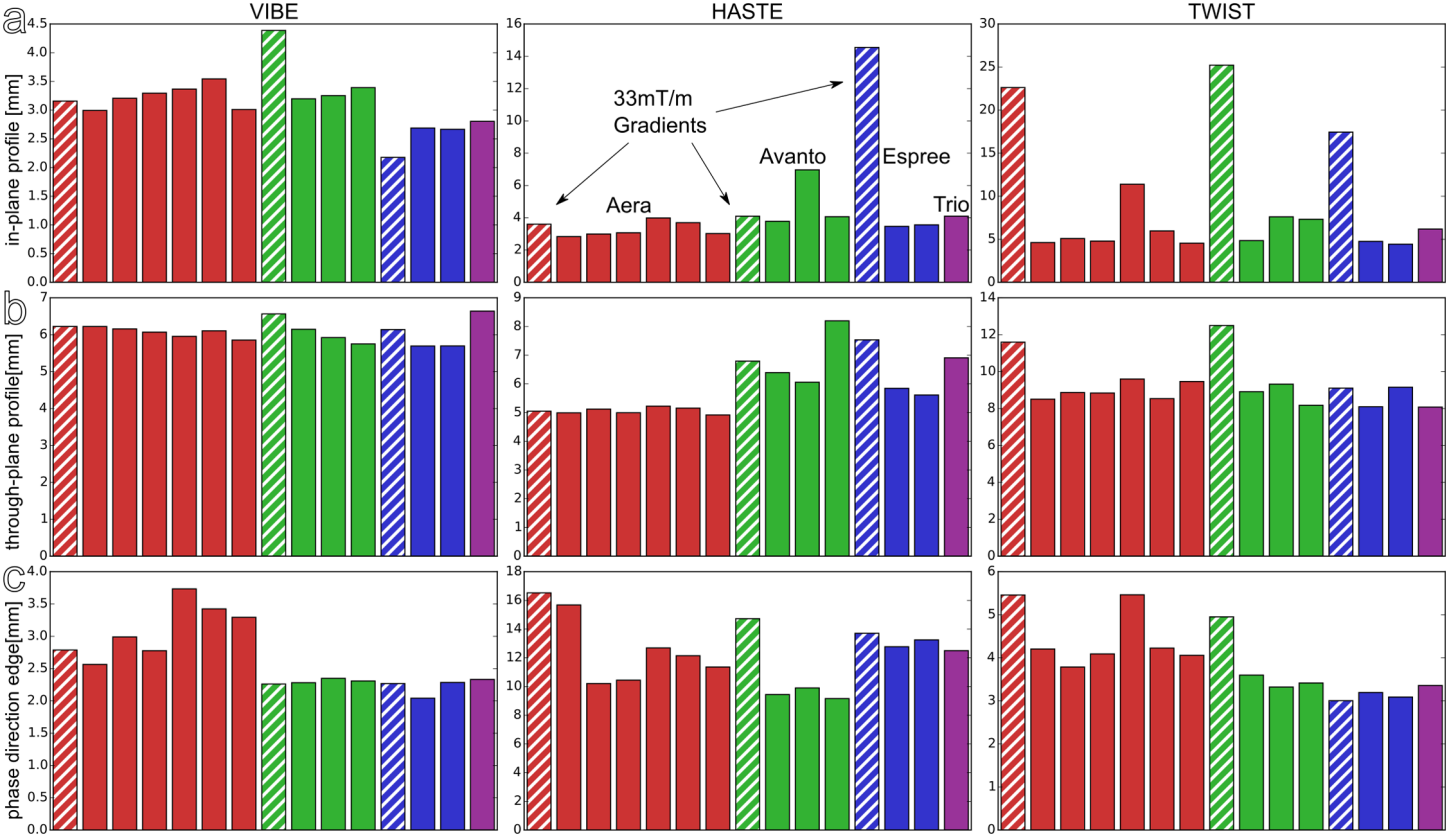
In-plane (a) and through-plane (b) profile widths detected in three selected sequences, sorted by site and scanner type. While the actual diameter of the rod is 6mm, these values are derived from the FWHM of a fitted function and thus should be smaller. (c) Shows the edge widths detected at the muscle-equivalent compartment in phase encoding direction. Hatched bars represent MR scanners with 33mT/m gradient systems. Please note that only the phantom measurements acquired at baseline of the *in vivo* study are displayed here.

Fig 4 shows the profile widths detected in three selected sequences, reflecting the depiction of structures: The VIBE, HASTE and TWIST measurements, all acquired in coronal orientation. The values represent a single time point (measurements performed at baseline of the *in vivo* study) and are ordered by the different scanner types used. The upper and centre rows contain the detected apparent width of the acrylic rod in the phantom, both within the imaging plane and perpendicular to it (through-plane). Again, the statistical tests for the difference between the scanner types were applied on all available measurements (i.e. baseline measurements and subsequent follow-up measurements at 3 month intervals), yielding p<0.05 only for the difference between the Espree and Aera, as well as Espree and Avanto. Similarly, structure and edge representation measures in the other sequences and directions were found to be significantly different among several of the scanner types in use.

Average edge widths were found to range from (1.79±0.71)mm in frequency- and (2.83±0.50)mm in phase-encoding direction in the coronal VIBE to (1.49±0.70)mm and (11.4±1.7)mm in coronal HASTE images. Regarding structural depiction derived from the apparent width of the acrylic rod, the difference between 45mT/m and 33mT/m gradient systems was notable, with the largest discrepancy of, on average, (4.1±2.3)mm to (7.7±4.0)mm, which was significant at p = 0.02 in the combined analysis of baseline and follow-up measurements.

## Discussion

Quantitative lung imaging in a multicentre environment warrants standardization of image quality as far as possible. This is addressed in all current multicentre studies with CT. Standardization procedures are constantly refined for example by the Quantitative Imaging Biomarkers Alliance (QIBA) [32], the SPIROMICS initiative [33], or SCIFI CF (Standardised Chest Imaging Framework for Interventions and Personalised Medicine in CF) [14]. While the measurements discussed here were being performed, the SPIROMICS initiative suggested to use standardized imaging protocols with additional scanning of standardized test objects (phantoms) at regular intervals to ensure protocol consistency and estimate comparability of image-based biomarkers across multiple centres. With regard to MRI in the lungs, methods to standardize image quality across multiple scanners are less developed, and commercially available reference phantoms focus on tissues with completely different signal characteristics than lung tissue [15–19]. Due to a lack of commercially available products suited specifically for morphological and functional *in vivo* ^1^H-MRI of the lung in multicentre patient studies, we aimed to design and apply a dedicated MRI reference phantom to detect and quantify diverging SNR and CNR of lung and chest tissue components with different MRI scanner models. This bears relevance, since heterogeneous equipment with diverging design and software is nearly inevitable when performing imaging in large *in vivo* multicentre studies as observed in multicentre CT [13, 34] and MRI [8, 24] based lung imaging studies, potentially influencing assessment and interpretation imaging data/biomarkers. With regard to multicentre MRI and also CT it is nearly impossible to adjust the various scan parameters in a way that scanners of different design and software provide completely identical image characteristics. Especially in MR imaging, it would mean to adjust image quality towards the level of the least capable scanners. This is important since the lungs provide only poor signal, and it is difficult to obtain images with sufficient quality to discriminate pulmonary physiology and pathology. Consequently, current MRI studies usually define a base protocol which is modified to the capabilities of individual MRI scanners, instead of maximizing effective inter-scanner variability on the cost of SNR. Such adjustments of the base protocol can also contribute to heterogeneity and have to be addressed together with other previously mentioned determinants of image quality.

Since lung MRI is complicated by extremely short T2* relaxation times [22] and very low proton density, only comparatively low spatial resolutions are achievable in lung MRI. Accordingly, the design of our phantom was not focused on the depiction of small details, thus allowing for a cost-effective design. Since the necessity of having measurements performed by technicians on site using the given study protocol is a limiting factor, the design shown and examined here also prioritizes ease of use over precision.

Repeat measurements for short-term intra-scanner variability showed a fairly good reproducibility of the measurement of one phantom instance on the same scanner with variabilities below 5%. Due to limited precision in the manufacturing process, the inter-instance variability (of the 12 instances produced) on this scanner is slightly larger, between 3.2% and 12.2%. Still, the individual phantom instances were considered sufficiently comparable to observe inter-scanner variability across multiple centres. The measures of image quality determined using the phantoms display substantial differences between the examined scanner systems at the respective study centres, both among scanners of the same type and, more noticeably, between different scanner types. Importantly, for most of the MRI sequences, SNR measurements of the phantom´s lung compartment showed far higher inter-scanner variability compared to the muscle, fat or blood compartment. Notably, the divide between the Aera systems and other scanners visible in Figs 3 and 4 reflects not only the physical characteristics of the systems, but also the different generations of the software running on the scanners. Additionally, note that the signal and noise distributions gained from each compartment can also be used to determine contrast-to-noise ratios between each pair of compartments, as reported in the supplement (see S1 Text).

While the profile widths shown in Fig 4 primarily reflect the difference between the available scanner software versions, the through-plane profiles also highlight the effect of the gradient system available on each scanner. Since lung MRI relies on short echo times to maximize signal, gradients are used to their full extent. Since the TWIST measurement is used for dynamic contrast enhanced perfusion analysis, high temporal resolutions and thus fast measurement times are relevant as well, requiring the use of coarser resolutions on scanners with weak gradient systems, which is well recognized by increased through-plane profile width as observed in the phantom measurements.

Since, as stated above, the difficulties of lung imaging lead to optimizing acquisition parameters for image quality rather than comparability, both image resolutions and parallelization ratios differ significantly between the scanners models in use. Accordingly, the calculated profile widths and SNR values alone do not provide a complete measure of image quality, but should provide sufficient quantitative information to estimate the variability of image quality.

By analogy to previous multicentre CT studies with pulmonary imaging, we observed substantial inter-scanner variability in a typical setting of multicentre lung MRI. Other research groups who were concerned with image quality in multicentre MRI studies focused on other organs than the lungs, but also observed substantial inter-scanner variability: Colombo et al. investigated the variability of 12 MRI systems from 0.5T to 1.5T in a multicentre setup. They investigated short- and mid-term variability of SNR and image uniformity (U%) using a spin echo sequence and a simple 20–24 cm phantom body filled with paramagnetic fluid (relaxation time in clinical range). Imaging of the phantom was repeated at baseline and 24h later and subsequently twice a week for 5 weeks. SNR and U% values showed significant differences among the scanner units. The results of the 24h measurements did not show significant heterogeneity during daily time interval for all the devices. In longitudinal measurements they observed a variability of 3% of the reference level for both parameters [35].

Belli et al. designed and applied a cylindrical doped water reference phantom for multicentre comparison of different MR scanners for quantitative diffusion-weighted imaging in twenty-six imaging facilities using 35 MR scanners with field strengths between 1T and 3T. Mean apparent diffusion coefficient (ADC) values were calculated and a significant difference between 1.5T and 3T was observed for high b-values. Short-term repeatability of ADC measurement (5 measurements, 1 minute delay each) was found <2.5% for all MR scanners [36].

In the US, the wide-spread MRI accreditation program of the American College of Radiology (ACR) evaluates the qualifications of personnel, the quality control program, MRI safety policies and image quality [37]. The accreditation test requires the acquisition of phantom images, and 2 different MRI phantoms are available from the ACR depending on the scanner unit. Employing an ACR phantom, Fu et al. investigated inter-site variability in SNR, image uniformity, width and height [17]. Mean SNR, image uniformity, width and height showed significant variability among the different scanners.

Harmonizing lung imaging biomarkers across multiple centres is challenging with CT, but is even more complicated with MRI, not least as the MR signal itself is not normalized to physical tissue characteristics as are density values in CT. Reference phantoms as demonstrated in the present study allow for a rough but objective estimation of inter-scanner variability, opening up several opportunities for handling inter-scanner inconsistencies: One possible way would be to test the scanners at potential imaging sites already in the planning stage of a clinical multicentre study with appropriate phantoms and exclude scanners or even imaging sites which show substantial downside deviation or do not reach a certain pre-defined quality threshold. Notably, phantom measurements cannot improve images of insufficient quality. In this process, the focus should be on image quality related parameters which are most critical for the respective *in vivo* study. Not only main field strength, but also gradient system, software version (which in this case also determines receive coils), bore dimensions and number of available receive channels should be considered.

When performing *in vivo* imaging within large networks or cohort studies (as in the present study), it is a common problem that imaging centres with heterogeneous equipment have to be included. In such cases, the true extent and underlying causes of inter-centre variability of imaging biomarkers are a matter of speculation, unless phantom measurements are available providing objective measures of variability. Differences in scanner hard- or software should be regarded as the underlying cause of such inconsistencies if MRI biomarkers (for example emphysema score) and phantom measurements (for example SNR) are strongly correlated and if both show a high variability between different centres. This could potentially bias comparison of imaging biomarkers with clinical tests, for example when correlating emphysema or perfusion scores and genetics. In such cases, *in vivo* datasets from scanners with substantial (downside) deviation compared to the mean of all available scanners could be excluded from statistical analyses, if relevant. However, this is certainly not cost- or time effective. As alternative approach with comparatively low drop-out rate, a standardization of biomarkers using z-transform can be attempted, but it requires normally distributed data and large sample size. Data harmonisation of imaging biomarkers using other mathematical procedures such as regression models or machine learning algorithms based on quantitative phantom measures are still of experimental character. Concerning quantitative lung perfusion biomarkers such as the pulmonary blood flow, absolute numbers of such perfusion indices could be replaced by measures of regional perfusion heterogeneity (variance), which should be less susceptible to inter-scanner variability. The limitations of the present reference phantom for the depiction of lung MRI measurements are significant: While lung signal is strongly influenced by the flow of blood, the phantom does not implement simulated perfusion. In particular, the contrast-agent based dynamic perfusion measurement included in the study protocol cannot be fully addressed this way. Of course, implementing this in a phantom would be more expensive and complex. Further, due to the compact design the phantom reflects the influence of different bore-sizes or macroscopic field homogeneity only to a limited degree [38]. Macroscopic field homogeneity is of relevance in lung imaging, and can only be addressed with phantoms of adequate size (app. 50cm×50cm×40cm), ideally with anatomically shaped units. Unfortunately, the mass and material cost of a phantom grows with the 8th power of each dimension, assuming the same construction as in the present study. In consequence, high costs as well as difficulty with production, transport, storage and handling would have to be expected. However, lighter or cheaper components are still not available for MRI phantom production. As we could not find a reasonable option to cover the entire relevant Field of View, in this work we chose to focus on the signal characteristics in the isocentre of the scanner. By analogy, contemporary established CT reference phantoms such as the COPDGene2 test-object [39] or common quality assurance phantoms such as the CatPhan [40] also cover only a small field-of-view, while inhomogeneity of SNR throughout the FOV can also be found in CT imaging [41, 42].Determining image noise for SNR calculations accurately from a single MR measurement on its own is challenging as described above in the methods section. Simply put, because of the coil sensitivities and the image reconstruction process, image noise is very low, with markedly heterogeneous distribution, while the noise of the individual coils is correlated with each other. For a more accurate noise measurement, the extent of noise of the individual coils would have to be assessed, and measurements without excitation pulses would have to be performed. However, both is too complex to be achieved at multiple centres with trained technicians performing measurements at 3 month intervals. Image characteristics of the 3T scanner tended to be different from the other scanners. However, statistical significance could not be found since there was only one such scanner included in this study with a small number of measurements. Nonetheless, we considered it noteworthy due to the novelty of the study subject. Besides, the present study included only scanners from a single manufacturer since the scanners were employed in the overarching *in vivo* study for which the phantom was designed. Hence, inter-vendor variability could not be investigated. The monitored timespan does not yet provide enough data to confirm that the phantoms’ MRI parameters remain constant over several years, which would be long enough for applicability in a potential long-term follow-up study. Nevertheless, the phantom experiments shown support a notable influence of the choice of MRI scanners on the homogeneity of image quality in the multicentre study. Given the very wide range of scanner models employed and investigated here, the validation of the phantom should be applicable for other multicentre lung MR studies. Further development will be necessary to improve accuracy and allow for a profound assessment of functional MRI acquisitions.

### Conclusion

The presented reference phantom allows to reproducibly quantify relevant metrics of image quality in multicentre *in vivo* studies using contemporary proton MRI protocols for lung imaging. Even when making efforts to create a fairly uniform scanner assortment suitable for lung imaging, substantial inter-centre variability is inevitable when employing scanner types with diverging design or software, and can even be found among the same scanner models due different hard- and software specifications. Besides, objective measures of inter-scanner variability can only be achieved with reference phantoms specifically designed for lung MRI, and the data can be helpful to detect or maybe overcome bias in the interpretation of study results. Finally, comprehensive standardization of lung MRI biomarkers remains a challenge.

## Supporting information

S1 Table Device attributes of the scanner models used. (PDF)

S2 Table Sequence parameters on 4 different scanner models. Note that for all models except the Trio there is an additional modified protocol for the version with weaker gradient system, which requires different TE and TR.
(PDF)

S3 Table CNR between the individual phantom compartments. These are (measured across all 15 scanners with 12 phantom instances) at study baseline. This is the mean and standard deviation of the median CNR found in each measurement.
(PDF)

S4 Table Signal ratios between the individual compartments, analogous to S3 Table. (PDF)

S1 Fig Examples of profile and edge depiction quantification. a,b: To determine profile depiction, signal parallel (**a**) to the 6mm acrylic rod’s passage through slices and orthogonal (**b**) to it is fitted with a gauss curve in each slice where it is visible. The full width half maximum of the curve is used as a measure of structural depiction, averaged over all slices. Only one slice of the coronal HASTE is shown here. c,d: To determine edge widths, an offset sigmoidal function α/(1+exp((x-β)/γ))+δ is fitted to the signal perpendicular to the edges in the image. γ is used as a measure of edge blurring, while the other fit parameters are discarded. Note the shape of the edge in the muscle compartment (short T2) in **c** and **d**.
(SVG)

S1 Text Additional details on scanner properties, image data analysis and contrast-to-noise. (PDF)

## References

[1] Wielpütz MO, Eichinger M, Biederer J, Wege S, Stahl M, Sommerburg O, et al Imaging of Cystic Fibrosis Lung Disease and Clinical Interpretation. Fortschr Röntgenstr. 2016;188(09):834–45. 10.1055/s-0042-104936 27074425

[2] Stahl M, Wielpütz DMO, Graeber DSY, Joachim MC, Sommerburg DO, Kauczor PH-U, et al Comparison of Lung Clearance Index and Magnetic Resonance Imaging for Assessment of Lung Disease in Children With Cystic Fibrosis. American Journal of Respiratory and Critical Care Medicine. 2017;0(ja):null–null. 10.1164/rccm.201604-0893OC 27575911

[3] Wielpütz MO, Mall MA. Imaging modalities in cystic fibrosis: emerging role of MRI. Current opinion in pulmonary medicine. 2015;21(6):609–16. doi: 10.1097/MCP.0000000000000213 26390331 10.1097/MCP.0000000000000213

[4] Ohno Y, Iwasawa T, Seo JB, Koyama H, Takahashi H, Oh Y-M, et al Oxygen-enhanced magnetic resonance imaging versus computed tomography: multicenter study for clinical stage classification of smoking-related chronic obstructive pulmonary disease. American journal of respiratory and critical care medicine. 2008;177(10):1095–102. doi: 10.1164/rccm.200709-1322OC 18276941 10.1164/rccm.200709-1322OC

[5] Hueper K, Parikh M, Prince MR, Schoenfeld C, Liu C, Bluemke DA, et al Quantitative and Semi-quantitative Measures of Regional Pulmonary Parenchymal Perfusion by Magnetic Resonance Imaging and their Relationships to Global Lung Perfusion and Lung Diffusing Capacity—The MESA COPD Study. Investigative radiology. 2013;48(4):223-. doi: 10.1097/RLI.0b013e318281057d 23385398 10.1097/RLI.0b013e318281057d PMC3952075

[6] Hueper K, Vogel-Claussen J, Parikh MA, Austin JHM, Bluemke DA, Carr J, et al Pulmonary microvascular blood flow in mild chronic obstructive pulmonary disease and emphysema. The MESA COPD Study. American journal of respiratory and critical care medicine. 2015;192(5):570–80. doi: 10.1164/rccm.201411-2120OC 26067761 10.1164/rccm.201411-2120OC PMC4595687

[7] Kirby M, Pike D, McCormack DG, Lam S, Sin DD, Coxson HO, et al Longitudinal computed tomography and magnetic resonance imaging of COPD: Thoracic Imaging Network of Canada (TINCan) study objectives. Chronic Obstructive Pulmonary Diseases: Journal of the COPD Foundation. 2014;1(2):200–11. doi: 10.15326/jcopdf.1.2.2014.0136 28848822 10.15326/jcopdf.1.2.2014.0136 PMC5556865

[8] CTEPH DIAGNOSIS Europe—MRI. https://clinicaltrials.gov/show/NCT02791282.

[9] Bamberg F, Kauczor H-U, Weckbach S, Schlett CL, Forsting M, Ladd SC, et al Whole-body MR imaging in the German National Cohort: rationale, design, and technical background. Radiology. 2015;277(1):206–20. doi: 10.1148/radiol.2015142272 25989618 10.1148/radiol.2015142272

[10] Biederer J, Mirsadraee S, Beer M, Molinari F, Hintze C, Bauman G, et al MRI of the lung (3/3)—current applications and future perspectives. Insights into imaging. 2012;3(4):373–86. doi: 10.1007/s13244-011-0142-z 22695943 10.1007/s13244-011-0142-z PMC3481076

[11] Biederer J, Beer M, Hirsch W, Wild J, Fabel M, Puderbach M, et al MRI of the lung (2/3). Why, when, how? Insights into Imaging. 2012;3(4):355–71. doi: 10.1007/s13244-011-0146-8 22695944 10.1007/s13244-011-0146-8 PMC3481084

[12] Wild JM, Marshall H, Bock M, Schad LR, Jakob PM, Puderbach M, et al MRI of the lung (1/3): methods. Insights into Imaging. 2012;3(4):345–53. doi: 10.1007/s13244-012-0176-x 22695952 10.1007/s13244-012-0176-x PMC3481083

[13] Regan EA, Hokanson JE, Murphy JR, Make B, Lynch DA, Beaty TH, et al Genetic epidemiology of COPD (COPDGene) study design. COPD: Journal of Chronic Obstructive Pulmonary Disease. 2011;7(1):32–43. 10.3109/15412550903499522 PMC2924193 20214461

[14] Kuo W, Kemner-van de Corput MPC, Perez-Rovira A, de Bruijne M, Fajac I, Tiddens HAWM, et al Multicentre chest computed tomography standardisation in children and adolescents with cystic fibrosis: the way forward. European Respiratory Journal. 2016. 10.1183/13993003.01601-2015 27076593

[15] Chen Q, Jakob P, Griswold M, Levin D, Hatabu H, Edelman R. Oxygen enhanced MR ventilation imaging of the lung. Magnetic Resonance Materials in Physics, Biology and Medicine. 1998;7(3):153–61. 10.1007/BF02591332 10050941

[16] Davids M, Zöllner FG, Ruttorf M, Nees F, Flor H, Schumann G, et al Fully-automated quality assurance in multi-center studies using MRI phantom measurements. Magnetic Resonance Imaging. 2014;32(6):771–80. doi: 10.1016/j.mri.2014.01.017 24602825 10.1016/j.mri.2014.01.017

[17] Fu L, Fonov V, Pike B, Evans AC, Collins DL. Automated Analysis of Multi Site MRI Phantom Data for the NIHPD Project. In: Larsen RNMSJ, editor.: Springer Berlin Heidelberg; 2006 p. 144–51. 10.1007/11866763_18 17354766

[18] Ihalainen TM, Lönnroth NT, Peltonen JI, Uusi-Simola JK, Timonen MH, Kuusela LJ, et al MRI quality assurance using the ACR phantom in a multi-unit imaging center. Acta Oncologica. 2011;50(6):966–72. doi: 10.3109/0284186X.2011.582515 21767198 10.3109/0284186X.2011.582515

[19] Mulkern RV, Forbes P, Dewey K, Osganian S, Clark M, Wong S, et al Establishment and Results of a Magnetic Resonance Quality Assurance Program for the Pediatric Brain Tumor Consortium. Academic Radiology. 2008;15(9):1099–110. doi: 10.1016/j.acra.2008.04.004 18692750 10.1016/j.acra.2008.04.004 PMC2561197

[20] Renvall V. Functional magnetic resonance imaging reference phantom. Magnetic resonance imaging. 2009;27(5):701–8. doi: 10.1016/j.mri.2008.11.007 19152772 10.1016/j.mri.2008.11.007

[21] Biederer J, Heussel CP, Puderbach M, Wielpuetz MO. Functional Magnetic Resonance Imaging of the Lung. Semin Respir Crit Care Med. 2014;35(01):074–82. 10.1055/s-0033-1363453 24481761

[22] Hatabu H, Alsop DC, Listerud J, Bonnet M, Gefter WB. T2* and proton density measurement of normal human lung parenchyma using submillisecond echo time gradient echo magnetic resonance imaging. Eur J Radiol. 1999;29(3):245–52. 10399610 10.1016/s0720-048x(98)00169-7

[23] Yu J, Xue Y, Song HK. Comparison of lung T2* during free-breathing at 1.5 T and 3.0 T with ultrashort echo time imaging. Magn Reson Med. 2011;66(1):248–54. doi: 10.1002/mrm.22829 21695727 10.1002/mrm.22829 PMC3122137

[24] Jobst BJ, Biederer J, Fellhauer I I., Triphan S, Burmester K, Schliebus JaA, et al Image-based structural and functional phenotyping of the German COPDcohort (COSYCONET) using MRI and CT. Insights into Imaging. 2015;6(1):444-.

[25] Rosato A, Strandburg KJ, Prinz F, Swendsen RH. Why the Brazil nuts are on top: Size segregation of particulate matter by shaking. Phys Rev Lett. 1987;58:1038–40. doi: 10.1103/PhysRevLett.58.1038 10034316 10.1103/PhysRevLett.58.1038

[26] Knight JB, Jaeger HM, Nagel SR. Vibration-induced size separation in granular media: The convection connection. Phys Rev Lett. 1993;70:3728–31. doi: 10.1103/PhysRevLett.70.3728 10053947 10.1103/PhysRevLett.70.3728

[27] Jobst BJ, Triphan S, Sedlaczek O, Anjorin A, Kauczor HU, Biederer J, et al Comparative assessment of T1 imaging, oxygen-enhanced MRI and first-pass perfusion MRI in chronic obstructive pulmonary disease at 1.5 Tesla. Insights Imaging. 2014;5:340-.

[28] Triphan SM, Breuer FA, Gensler D, Kauczor HU, Jakob PM. Oxygen enhanced lung MRI by simultaneous measurement of T1 and T2 * during free breathing using ultrashort TE. J Magn Reson Imaging. 2015;41(6):1708–14. doi: 10.1002/jmri.24692 . 25044618 10.1002/jmri.24692

[29] Triphan SM, Jobst BJ, Breuer FA, Wielputz MO, Kauczor HU, Biederer J, et al Echo time dependence of observed T1 in the human lung. J Magn Reson Imaging. 2015;42(3):610–6. doi: 10.1002/jmri.24840 . 25604043 10.1002/jmri.24840

[30] Gonzalez RC, Woods RE. Digital image processing. Upper Saddle River, NJ: Prentice Hall; 2012.

[31] Triphan SM, Jobst BJ, Anjorin A, Sedlaczek O, Wolf U, Terekhov M, et al Reproducibility and comparison of oxygen-enhanced T1 quantification in COPD and asthma patients. PLoS One. 2017;12(2):e0172479 doi: 10.1371/journal.pone.0172479 . 28207845 10.1371/journal.pone.0172479 PMC5312969

[32] Chen-Mayer HH, Fuld MK, Hoppel B, Judy PF, Sieren JP, Guo J, et al Standardizing CT lung density measure across scanner manufacturers. Medical physics. 2017;44(3):974–85. doi: 10.1002/mp.12087 28060414 10.1002/mp.12087 PMC6276120

[33] Sieren JP, Newell JD Jr, Barr RG, Bleecker ER, Burnette N, Carretta EE, et al SPIROMICS protocol for multicenter quantitative computed tomography to phenotype the lungs. American journal of respiratory and critical care medicine. 2016;194(7):794–806. doi: 10.1164/rccm.201506-1208PP 27482984 10.1164/rccm.201506-1208PP PMC5074650

[34] Agusti A, Calverley PM, Celli B, Coxson HO, Edwards LD, Lomas DA, et al Characterisation of COPD heterogeneity in the ECLIPSE cohort. Respir Res. 2010;11:122 doi: 10.1186/1465-9921-11-122 . 20831787 10.1186/1465-9921-11-122 PMC2944278

[35] Colombo P, Baldassarri A, Del Corona M, Mascaro L, Strocchi S. Multicenter trial for the set-up of a MRI quality assurance programme. Magnetic resonance imaging. 2004;22(1):93–101. doi: 10.1016/j.mri.2003.04.001 14972398 10.1016/j.mri.2003.04.001

[36] Belli G, Busoni S, Ciccarone A, Coniglio A, Esposito M, Giannelli M, et al Quality assurance multicenter comparison of different MR scanners for quantitative diffusion-weighted imaging. Journal of Magnetic Resonance Imaging. 2016;43(1):213–9. doi: 10.1002/jmri.24956 26013043 10.1002/jmri.24956

[37] Shu Y, Gorny KR, Felmlee JP, Pooley RA, Edmonson HA. Practical considerations for ACR MRI accreditation. Journal of the American College of Radiology. 2014;11(1):94–6. doi: 10.1016/j.jacr.2013.09.024 24387966 10.1016/j.jacr.2013.09.024

[38] Torfeh T, Hammoud R, McGarry M, Al-Hammadi N, Perkins G. Development and validation of a novel large field of view phantom and a software module for the quality assurance of geometric distortion in magnetic resonance imaging. Magnetic resonance imaging. 2015;33(7):939–49. doi: 10.1016/j.mri.2015.04.003 25882440 10.1016/j.mri.2015.04.003

[39] Sieren JP, Hoffman EA, Fuld MK, Chan KS, Guo J, Newell JD Jr. Sinogram Affirmed Iterative Reconstruction (SAFIRE) versus weighted filtered back projection (WFBP) effects on quantitative measure in the COPDGene 2 test object. Med Phys. 2014;41(9):091910 doi: 10.1118/1.4893498 . 25186397 10.1118/1.4893498 PMC4149690

[40] Gulliksrud K, Stokke C, Martinsen AC. How to measure CT image quality: variations in CT-numbers, uniformity and low contrast resolution for a CT quality assurance phantom. Phys Med. 2014;30(4):521–6. doi: 10.1016/j.ejmp.2014.01.006 . 24530005 10.1016/j.ejmp.2014.01.006

[41] Flohr TG, Stierstorfer K, Ulzheimer S, Bruder H, Primak AN, McCollough CH. Image reconstruction and image quality evaluation for a 64-slice CT scanner with z-flying focal spot. Med Phys. 2005;32(8):2536–47. doi: 10.1118/1.1949787 . 16193784 10.1118/1.1949787

[42] Bamba J, Araki K, Endo A, Okano T. Image quality assessment of three cone beam CT machines using the SEDENTEXCT CT phantom. Dentomaxillofac Radiol. 2013;42(8):20120445 doi: 10.1259/dmfr.20120445 . 23956235 10.1259/dmfr.20120445 PMC3922264

