# Supplementary material for: Design and application of an MR reference phantom for multicentre lung imaging trials
Source: PLoS One. 2018 Jul 5;13(7):e0199148. doi: 10.1371/journal.pone.0199148 (PMC6033396; doi:10.1371/journal.pone.0199148)
Supplement: S1 Table — (PDF) [file pone.0199148.s001.pdf]

model	soft-ware version	gradient strength [mT/m]	channels	B0 [T]	bore diam. [cm]	bore length [cm]	No used
Aera	VE11	33	48	1.5	70	137	1
Aera	VD13	45	48	1.5	70	137	6
Avanto	VB17	33	8	1.5	60	150	1
Avanto	VB17	45	32	1.5	60	150	2
Avanto	VD13	45	32	1.5	60	150	1
Espree	VB19	33	32	1.5	70	120	1
Espree	VB17	45	32	1.5	70	120	2
Trio	VB17	45	32	3.0	60	198	1
