# Supplementary material for: Design and application of an MR reference phantom for multicentre lung imaging trials
Source: PLoS One. 2018 Jul 5;13(7):e0199148. doi: 10.1371/journal.pone.0199148 (PMC6033396; doi:10.1371/journal.pone.0199148)
Supplement: S2 Table — Note that for all models except the Trio there is an additional modified protocol for the version with weaker gradient system, which requires different TE and TR. (PDF) [file pone.0199148.s002.pdf]

model/ sequence name	TE [ms]	TR [ms]	voxel size [mm²]	slice thick. [mm]	matrix size	flip angle [°]	orien-tation	iPAT factor
Aera								
Angio FLASH	1.04	2.80	1.0×1.0	1.8	336×384	25.0	cor	3
BLADE	73.00	905.00	1.2×1.2	6.0	320×320	144.0	cor	2
HASTE	20.00	314.00	0.8×0.8	6.0	512×512	140.0	cor	3
HASTE	27.00	500.00	1.4×1.4	8.0	320×260	150.0	tra	2
HASTE IRM	72.00	502.00	1.6×1.6	6.0	256×256	180.0	tra	2
TrueFISP	1.17	448.93	0.8×0.8	4.5	512×512	66.0	cor	3
TWIST	0.76	1.73	1.8×1.8	5.0	208×256	20.0	cor	2
VIBE	1.63	3.61	1.4×1.4	4.0	288×288	5.0	cor	2
VIBE	1.61	3.29	1.2×1.2	4.0	320×240	5.0	tra	2
VIBE FS	1.61	3.29	1.2×1.2	4.0	320×240	5.0	tra	2
Avanto								
Angio FLASH	0.93	2.58	1.3×1.3	1.8	324×384	25.0	cor	2
BLADE	121.00	2040.00	1.5×1.5	6.0	320×320	150.0	cor	2
HASTE	29.00	396.00	1.8×1.8	6.0	256×256	180.0	cor	3
HASTE	27.00	422.00	1.8×1.8	8.0	256×192	180.0	tra	2
HASTE IRM	80.00	1400.00	1.3×1.3	8.0	384×348	150.0	tra	2
TrueFISP	1.13	406.68	0.9×0.9	4.5	512×512	70.0	cor	2
TWIST	0.70	1.78	2.0×2.0	5.0	208×256	20.0	cor	2
VIBE	1.04	3.00	1.0×1.0	4.0	512×512	5.0	cor	2
VIBE	1.15	3.23	0.9×0.9	4.0	512×384	5.0	tra	2
VIBE FS	1.15	3.30	0.9×0.9	4.0	512×384	8.0	tra	2
Espree								
Angio FLASH	0.96	2.46	1.2×1.2	1.8	324×384	25.0	cor	2
BLADE	132.00	2040.00	1.4×1.4	6.0	320×320	150.0	cor	2
HASTE	31.00	540.00	1.8×1.8	6.0	256×256	180.0	cor	2
HASTE	22.00	422.00	1.8×1.8	8.0	256×192	180.0	tra	2
HASTE IRM	79.00	1400.00	1.2×1.2	8.0	384×348	150.0	tra	2
TrueFISP	1.14	419.51	0.9×0.9	4.5	512×512	70.0	cor	2
TWIST	0.74	1.87	1.8×1.8	5.0	208×256	20.0	cor	2
VIBE	1.11	3.06	0.9×0.9	4.0	512×512	5.0	cor	2
VIBE	1.13	3.00	0.9×0.9	4.0	512×384	5.0	tra	2
VIBE FS	1.19	3.17	0.9×0.9	4.0	512×384	8.0	tra	2
Trio								
Angio FLASH	0.93	2.58	1.3×1.3	1.8	324×384	16.0	cor	2

BLADE	131.00	2040.00	1.5×1.5	8.5	320×320	120.0	cor	2
HASTE	36.00	396.00	1.8×1.8	6.0	256×256	140.0	cor	3
HASTE	28.00	452.00	1.8×1.8	8.0	256×192	140.0	tra	2
HASTE IRM	80.00	1400.00	1.3×1.3	8.0	384×348	150.0	tra	2
TrueFISP	1.13	406.68	0.9×0.9	4.5	512×512	32.0	cor	2
TWIST	0.70	1.78	2.0×2.0	5.0	208×256	12.0	cor	2
VIBE	0.78	2.80	1.0×1.0	4.0	512×512	5.0	cor	2
VIBE	0.87	3.00	0.9×0.9	4.0	512×384	5.0	tra	2
VIBE FS	0.87	3.00	0.9×0.9	4.0	512×384	8.0	tra	2
