# Supplementary material for: Design and application of an MR reference phantom for multicentre lung imaging trials
Source: PLoS One. 2018 Jul 5;13(7):e0199148. doi: 10.1371/journal.pone.0199148 (PMC6033396; doi:10.1371/journal.pone.0199148)
Supplement: S3 Table — These are (measured across all 15 scanners with 12 phantom instances) at study baseline. This is the mean and standard deviation of the median CNR found in each measurement. (PDF) [file pone.0199148.s003.pdf]

sequence	lung - blood	lung - muscle	lung - fat	blood - muscle	blood - fat	muscle - fat
VIBE cor	22.3 ±10.7	29.6 ±15.8	42.7 ±21.4	6.5 ±5.3	19.5 ±11.6	10.2 ±7.2
VIBE tra	22.4 ±10.1	29.5 ±14.6	45.7 ±17.7	7.1 ±5.2	21.2 ±8.7	6.2 ±6.5
HASTE cor	191.3 ±81.5	55.4 ±24.3	142.0 ±57.2	131.8 ±59.3	45.4 ±25.1	82.5 ±35.0
HASTE tra	181.3 ±89.8	56.0 ±32.8	116.2 ±65.4	129.9 ±61.1	56.3 ±26.4	65.4 ±37.2
TrueFISP	83.5 ±53.8	18.5 ±19.4	49.2 ±23.5	65.1 ±40.8	33.2 ±35.7	25.7 ±10.9
BLADE	132.0 ±80.1	8.8 ±3.7	63.5 ±23.6	124.1 ±78.3	71.1 ±42.4	54.5 ±20.9
HASTE IRM	112.3 ±29.9	9.1 ±6.2	2.0 ±2.2	105.1 ±26.8	115.0 ±30.4	9.9 ±6.9
Angio FLASH	1.2 ±1.4	3.0 ±2.6	13.3 ±3.4	1.8 ±1.2	12.0 ±2.2	10.0 ±1.6
TWIST	4.3 ±3.2	8.0 ±6.1	34.8 ±30.6	3.9 ±3.0	25.8 ±27.7	21.0 ±25.1
VIBE FS	23.7 ±7.6	28.8 ±14.2	9.3 ±6.6	5.5 ±7.6	16.8 ±6.5	23.5 ±11.4
