# Supplementary material for: Design and application of an MR reference phantom for multicentre lung imaging trials
Source: PLoS One. 2018 Jul 5;13(7):e0199148. doi: 10.1371/journal.pone.0199148 (PMC6033396; doi:10.1371/journal.pone.0199148)
Supplement: S4 Table — (PDF) [file pone.0199148.s004.pdf]

Signal Ratio	lung/blood	lung/muscle	lung/fat	blood/muscle	blood/fat	muscle/fat
VIBE cor	0.182 ±0.062	0.136 ±0.048	0.111 ±0.033	0.773 ±0.031	0.654 ±0.065	0.832 ±0.071
VIBE tra	0.177 ±0.058	0.136 ±0.053	0.108 ±0.033	0.771 ±0.063	0.642 ±0.047	0.810 ±0.078
HASTE cor	0.088 ±0.035	0.224 ±0.084	0.116 ±0.045	2.566 ±0.286	1.298 ±0.057	0.488 ±0.041
HASTE tra	0.106 ±0.036	0.222 ±0.089	0.127 ±0.049	2.336 ±0.306	1.336 ±0.067	0.564 ±0.060
TrueFISP	0.036 ±0.016	0.133 ±0.067	0.063 ±0.039	3.803 ±0.657	1.810 ±0.447	0.497 ±0.122
BLADE	0.029 ±0.022	0.203 ±0.128	0.032 ±0.019	11.373 ±5.129	1.962 ±0.747	0.150 ±0.026
HASTE IRM	0.036 ±0.018	0.250 ±0.165	1.149 ±1.338	8.695 ±2.975	27.759 ±21.390	2.900 ±3.102
Angio FLASH	0.500 ±0.108	0.271 ±0.066	0.081 ±0.016	0.562 ±0.060	0.163 ±0.044	0.294 ±0.072
TWIST	0.373 ±0.072	0.221 ±0.059	0.056 ±0.011	0.611 ±0.070	0.156 ±0.036	0.259 ±0.056
VIBE FS	0.131 ±0.051	0.083 ±0.046	0.353 ±0.188	0.812 ±0.105	3.464 ±1.649	5.378 ±1.799
