# Supplementary material for: Design and application of an MR reference phantom for multicentre lung imaging trials
Source: PLoS One. 2018 Jul 5;13(7):e0199148. doi: 10.1371/journal.pone.0199148 (PMC6033396; doi:10.1371/journal.pone.0199148)
Supplement: S1 Text — (PDF) [file pone.0199148.s006.pdf]

Additional details on scanner properties, image data analysis and contrast-to-noise ratios are given here.

Measurements

The four scanner models (Avanto, Aera, Espree and Trio) employed in both this and the *in vivo* study are from the same manufacturer, but differ in several categories: Aera, Avanto and Espree provide main fields of B_0=1.5T and the Trio has B_0=3.0T. The different scanner types show varying software versions, gradient strength, bore diameter and bore length and their receive amplifiers show different numbers of channels as displayed in S2 Table. In addition, the Aera has a more advanced set of receive coil arrays with a larger number of coils (body arrays with 18 rather than 6 coils, spine arrays with 32 rather than 18 coils). Besides, differences in gradient strength and software version can be found among scanners of the same type (Aera, Avanto, Espree).

The imaging protocol for the in vivo study was designed to provide diagnostic information on lung structure and function of COPD, and similar protocols have been previously used in cystic fibrosis. The base protocol was designed for the Siemens Aera scanner, and minor modifications hat to be made to me et the technical specifications of the Avanto, Espree and Trio scanners. Sequence parameters of the latter scanners are displayed in S3 Table.

Image data analysis

As explained in the main text, the shape of an acrylic rod as it appears in images and the reproduction of the phantom bottles' edges were calculated to reflect structural depiction. For this purpose, signal gradients orthogonal and parallel to the rod's orientation projected into the imaging slice are determined. These each correspond to the representation of the rod´s cross section in the slice and the slice profile, respectively. To reduce these curves to a single number, the Full Width at Half Maximum (FWHM) is calculated, as shown in S4 Fig. a&b.

Besides, the edge representation of each sequence is determined in two orthogonal directions within each image set. In this process, the centre of each compartment is calculated from its centre of mass and the signal gradient orthogonal to the two outside edges of the bottle, starting from this centre, is averaged in a small strip. Again, a single number for each direction is achieved by fitting a sigmoidal function to this gradient and using the width of the sigmoid as the measure of edge blurring. S4 Fig. c&d shows a representative example.

Results

As complement to the SNR values given in the main text, S5 Table contains the contrast-to-noise ratios (CNR) found on all scanners and S6 Table gives the ratio between the signals measured. The spread given here is the standard deviation between these measurements and thus reflects the difference between scanners (and phantom instances).
